# Dynamics of the straight-ahead preference in human visual cortex

**DOI:** 10.1007/s00429-019-01988-5

**Published:** 2019-12-02

**Authors:** Olena V. Bogdanova, Volodymyr B. Bogdanov, Jean-Baptiste Durand, Yves Trotter, Benoit R. Cottereau

**Affiliations:** 1 Centre de Recherche Cerveau et Cognition, Université de Toulouse, 31052 Toulouse, France; 2 Centre National de la Recherche Scientifique, 31055 Toulouse, France; 3 Centre de Recherche en Neurosciences de Lyon Inserm U1028-CNRS UMR5292 Bâtiment Inserm, 16 Avenue Doyen Lépine, 69676 Bron, France; 4 Laboratoire Génie Civil et Bâtiment, Universite de Lyon, Ecole Nationale des Travaux Publics de l’Etat, 3 rue Maurice Audin, 69518 Vaulx-en-Velin, France

**Keywords:** Straight-ahead, Visual cortex, EEG, Temporal dynamics

## Abstract

The objects located straight-ahead of the body are preferentially processed by the visual system. They are more rapidly detected and evoke stronger BOLD responses in early visual areas than elements that are retinotopically identical but located at eccentric spatial positions. To characterize the dynamics of the underlying neural mechanisms, we recorded in 29 subjects the EEG responses to peripheral targets differing solely by their locations with respect to the body. Straight-ahead stimuli led to stronger responses than eccentric stimuli for several components whose latencies ranged between 70 and 350 ms after stimulus onset. The earliest effects were found at 70 ms for a component that originates from occipital areas, the contralateral P1. To determine whether the straight-ahead direction affects primary visual cortex responses, we performed an additional experiment (*n* = 29) specifically designed to generate two robust components, the C1 and C2, whose cortical origins are constrained within areas V1, V2 and V3. Our analyses confirmed all the results of the first experiment and also revealed that the C2 amplitude between 130 and 160 ms after stimulus onset was significantly stronger for straight-ahead stimuli. A frequency analysis of the pre-stimulus baseline revealed that gaze-driven alterations in the visual hemi-field containing the straight-ahead direction were associated with a decrease in alpha power in the contralateral hemisphere, suggesting the implication of specific neural modulations before stimulus onset. Altogether, our EEG data demonstrate that preferential responses to the straight-ahead direction can be detected in the visual cortex as early as about 70 ms after stimulus onset.

**Electronic supplementary material:**

The online version of this article (10.1007/s00429-019-01988-5) contains supplementary material, which is available to authorized users.

## Introduction

The objects we are facing are endowed with a special behavioral status as they offer maximal affordance for manipulation but also represent potential obstacles during locomotion. Paying a special attention to these straight-ahead elements is thus desirable. Most of the time, our gaze is directed straight-ahead, so that the important neuronal resources allocated to central vision actually process these elements efficiently. However, it can happen that an unexpected event in the surrounding space attracts attention and consequently gaze direction and central vision toward an eccentric location. Elements located straight-ahead then fall in the periphery of the retina for which vision is much less accurate. Recently, a compensatory mechanism that permits an enhanced processing of the straight-ahead direction in this case has been evidenced by single-cell recordings in the primary visual (V1) area of rhesus macaque monkeys (Durand et al. 2010). Most V1 neurons with peripheral receptive fields (RF) exhibit an increased visual sensitivity as their RF is brought closer to the straight-ahead direction by progressively shifting gaze direction. In humans, a growing body of evidences also suggests that the straight-ahead direction benefits from a privileged processing in peripheral vision. Behavioral studies (Camors et al. 2016; Durand et al. 2012) showed that participants react faster to visual stimuli if they are located straight-ahead rather than in an eccentric position, even if their visual properties (i.e. their retinal images) are strictly identical. A recent functional imaging study established that in early visual cortex (i.e. in areas V1 and V2), the blood-oxygenation-level-dependent (BOLD) response is stronger for stimuli located along the straight-ahead direction (Strappini et al. 2015). It is tempting to interpret these human results as reflecting fast and low-level neural mechanisms similar to that evidenced in macaque. However, such an interpretation is hampered by the current lack of knowledge regarding the dynamics of the straight-ahead preference in humans.

Here, we address this issue with electroencephalographic (EEG) recordings in human participants, which permits to capture the dynamics of straight-ahead facilitation with a millisecond resolution. We recorded the event-related potentials (ERPs) in response to visually identical peripheral stimuli presented either straight-ahead or at an eccentric position of the egocentric space (i.e. relatively to the body). Visual ERPs include a number of task-specific components, reflecting different cortical processing stages (Di Russo et al. 2019). In a first experiment, we adapted the design of our previous behavioral experiments with peripheral stimuli located along the horizontal meridian (Camors et al. 2016; Durand et al. 2012). However, we used here large and high-contrast stimuli that are more efficient at evoking robust ERPs. The spatial configuration of the stimuli permitted to isolate components whose latencies ranged between 70 and 350 ms after stimulus onset. Because this experiment discovered earliest straight-ahead effects at 70 ms, we performed a second experiment specifically designed to test if these early effects originated from primary visual cortex. In this case, stimuli were presented within the four visual quadrants to elicit a C1 component. This EEG component arises around 60 ms after stimulus onset and is believed to reflect the first measurable feedforward responses from primary visual areas, i.e. V1 (Clark et al. 1995; Di Russo et al. 2003) but also V2 and V3, see (Ales et al. 2010). In addition, we tested the hypothesis that lateral gaze fixation might impact pre-stimulus onset activity in posterior sites to facilitate the processing of upcoming straight-ahead visual inputs.

## Materials and methods

### Subjects

Our study involved two groups of 29 subjects (15 men and 14 women in the first group, 21 men and 8 women in the second group), one for each EEG experiment. Most of these subjects were university students. They were all right-handed (or ambidextrous) with normal of corrected-to-normal vision. They all provided their written informed consent before participating in the study. The experimental protocol respected the Helsinki Declaration and was approved by a local ethics committee as a part of the OPTIVISION ANR research project.

### Stimuli

**Fig. 1:**
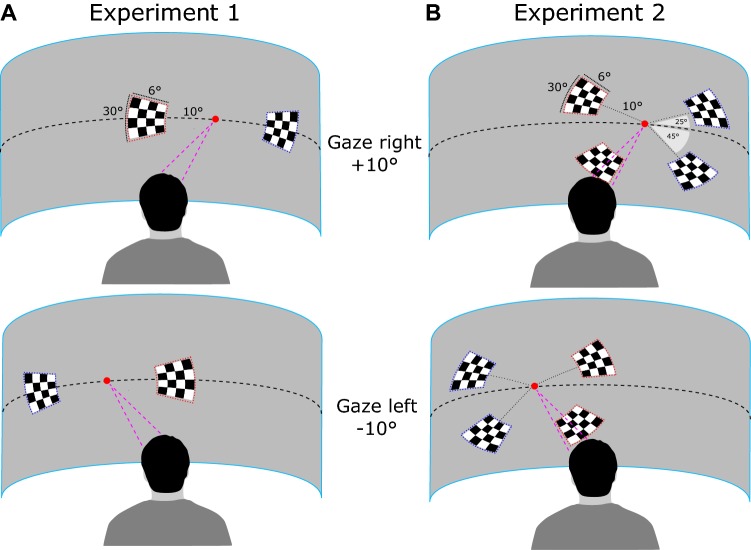
Experimental protocol. The red point indicates gaze fixation and is located 10° either leftward or rightward from the egocentric straight-ahead direction. **a** In the first experiment, the stimuli were displayed along the horizontal meridian (10° to left or to the right of the fixation point). **b** In the second experiment, they were located on the diagonals as originally proposed in (Di Russo et al. 2003; Miller et al. 2015). This configuration maximizes the amplitudes of the C1 and C2 components (see the details in the text). Straight-ahead and peripheral stimuli are, respectively, highlighted in red and blue

Our basic stimulus was a black and white checkerboard (100% of contrast) that spanned 30° of visual polar angle and ranged between 10 and 16° of eccentricity. This checkerboard contained 16 checks (4 × 4) of the same angular size. It was displayed on a gray convex screen, subtending 160° × 45° of visual angle at a viewing distance of 150 cm, using a video projector (NEC NP1250) set to run with 60 Hz refresh rate at 1400*1050 pixels resolution. The two experiments were controlled using the Psychophysics Toolbox extensions version 3.0 installed on Matlab^®^ R2011 (MathWorks, USA) software, running on an Intel Core i5 based computer. The two experiments differed solely in the locations of the stimuli. In the first experiment, we followed the experimental design of our previous behavioral study (Durand et al. 2012) and the stimulus was presented along the horizontal meridian (i.e. at eye level) at 10° of eccentricity either to the left or to the right of ocular fixation (Fig. 1a). In the second experiment, we modified this design to maximize the amplitude of the C1 component, which is believed to reflect the first EEG feedforward responses in early visual cortex (Clark et al. 1995). More specifically, we followed the approach described in two previous studies (Di Russo et al. 2003, 2012; Miller et al. 2015) by displaying the stimulus in one out of four positions relative to the point of ocular fixation. Two of these positions were localized in the upper visual field 25° above the horizontal meridian (i.e. above the eye level), 10° (either leftward or rightward) from fixation. The two others were localized in the lower visual field 45° below the horizontal meridian, 10° (either leftward or rightward) from fixation (Fig. 1b). These specific positions in the upper and lower visual field, respectively, activate opposite sides on the lower and upper banks of the calcarine sulcus (Di Russo et al. 2003). As a consequence, the corresponding evoked related potentials (ERPs) have inverted polarities and their subtraction permits to cancel-out all the other, not-polarity-inverting components as described in (Miller et al. 2015) and obtain C1 as well as the subsequent C2 component, which is believed to reflect feedback from extra-striate areas to early visual cortex.

### Experimental design

To get the EEG responses to alternative conditions that were visually identical but located at different egocentric spatial positions, relatively to the body (i.e. either along the straight-ahead direction or in the periphery), we manipulated gaze direction. In both experiments, we instructed participants to maintain their gaze on a red fixation point (6 arcmin of diameter) while the head orientation remained always aligned with the body axis. This point was located on the horizontal meridian at 10° either to the left or to the right of the straight-ahead direction (see Fig. 1).

The two gaze directions alternated every 20 presentations of the visual stimulus. Fixation stability with this paradigm was verified with an eye-tracker in a subset of subjects (see Supplementary Fig. 1 and the accompanying text). Participants were instructed to sit in a chair, legs uncrossed, hands on a table and faced a large and curved screen. Their head was placed on a head-support device clamped on top of the table and equipped with both chin and forehead supports. The uprights were further covered with sheets of dense foam to minimize head rotation during the experiment. The chair and head-support devices were positioned so as to ensure a fine alignment between the participants’ head and trunk axes. Participants were instructed to keep this position as constant as possible during the recordings. For each experiment, they performed four sessions of 120 stimuli that lasted about 4 min. Each stimulus was displayed during approximately 33 ms (two frames at 60 Hz). Within each session, stimuli were randomly presented either along with the straight-ahead direction (when gaze direction and azimuth were opposite) or at an eccentric position (when they were declined in the same direction (Fig. 1). Each session contained 6 blocks of 20 stimuli (3 for each gaze direction). Subjects were instructed to press a key with their right index whenever a stimulus appeared. This permitted to focus their attention on the stimuli and to refrain them from blinking during the blocks. The inter-stimulus interval (ISI) randomly varied between 1000 and 2200 ms to attenuate anticipation in the motor reports. Reaction times (RTs) were computed as the time elapsed between stimulus onset and button press. RTs shorter than 200 ms were considered as anticipatory responses and discarded from further analyses. RTs longer than 800 ms were considered as attentional lapses and excluded as well. For each subject, we computed the median RT for straight-ahead and eccentric stimulations. Then we computed for each subject the difference between the RTs corresponding to straight-ahead versus eccentric stimulations. The statistical significance of the effect was estimated using a bootstrap analysis performed with the STATISTICA 8 Software.

### EEG data recordings and pre-processing

EEG recordings were performed using a BioSemiActiveTwo system with active Ag/AgCl electrodes (BioSemi, Amsterdam, Netherlands). We collected data from 64 electrodes distributed over the scalp according to the 10–20 EEG system with a sampling rate of 2048 Hz. Electrolyte SIGNA gel was applied to the electrode caps for better conductance between electrodes and skin. The pre-processing of raw data and statistical analysis were conducted with custom scripts in Matlab using the Statistics and Machine Learning toolboxes (MathWorksinc). After removing the mean and regressing out any linear and one-phase sine and cosine trends for each session, data were high-pass filtered at 0.1 Hz with a fast Fourier transformation. Epochs were extracted from 600 ms before to 600 ms after stimulus onset, for a total duration of 1.2 s. To identify noisy/corrupted trials, we determine for each epoch the highest peak-to-peak amplitude across electrodes. When this value was higher than the 90th percentile of this measure among all the epochs of the same session, the epoch was considered as noisy and discarded. Therefore, about 10% of the trials were rejected with this process. All the data were then smoothed with a 10 ms moving average kernel.

During data analysis, the time-courses recorded on each of the electrodes of our individual subjects were plotted and visually inspected. We observed that, during baseline, a few channels (most of the time above frontal cortex) were systematically noisier than others. This noise was present independently of the experimental condition. Based on this empirical observation, we decided to perform an automated channel rejection at the group level to minimize the impact of these noisy data on further analyses. For each subject, we, therefore, removed the four noisiest electrodes. To find those electrodes, we first re-referenced the data using the average reference and estimated the difference between the minimum and maximum amplitudes of the global (i.e. across all conditions) response during the first 300 ms of the baseline. In most of the cases, these electrodes were localized above frontal cortex. As a consequence, this manipulation is rather unlikely to impact our results as our effects are mostly found in occipital and parietal electrodes. After this process, we re-referenced the data using the new grand average. Data were then baseline corrected using the 300 ms prior to stimulus onset.

### Statistical analysis

In our main analysis, time-locked event-related potentials (ERPs) were averaged separately according to stimulus position (left and right for the first experiment and upper-left, upper-right, lower-left and lower-right for the second experiment) and condition (i.e. whether the stimulus was presented along the straight-ahead direction or at an eccentric position). Our experiments were designed to elicit strong visual ERPs with amplitude peaks that are usually reported in EEG vision studies: P1, N1, and P2-P3 (and also the C1 and C2 for the second experiment). To characterize those peaks and to estimate their amplitude and latencies, we followed the general recommendations provided in (Luck 2005), we also used the methods described in (Di Russo et al. 2002) for the characterization of the early components (and of the associated topographies) evoked by peripheral visual stimulations. The extraction of the C1 and C2 components were based on the methods described in (Miller et al. 2015).

First, we characterized grand average peaks in mean waves and difference waves across straight-ahead and eccentric experimental conditions. In the first experiment with horizontal meridian stimulation we computed the difference between the responses to the left and the right hemifield stimulation to highlight contralateral P1 and N1 components (Di Russo et al. 2003). In the second experiment with quadrant stimulation we computed the difference between responses to upper and lower hemifield stimulation to assess C1 and C2 components (Di Russo et al. 2003; Miller et al. 2015). For both mean waves and difference waves we estimated global field power (GFP) at every time point as a standard deviation across electrodes (Skrandies 1990). The local peaks of GFP were used to define time-windows of interest (± 20 ms around the peak) that constrained our research of the components at the individual level.

*Individual peak amplitudes and latencies* Within these time-windows, we determined for each subject five electrodes with extreme amplitude deflection from the baseline. Time-courses on these five electrodes were first averaged. The peak of the resulting average time-course provided the amplitude and latency of the component. These data were included in the further statistical analysis only if the scalp topography at the time of the electrode cluster peak matched with the grand-average topography of the corresponding component. Otherwise, we considered that the signal-to-noise ratio (SNR) was insufficient and the corresponding data were not included in further analyses. Topographies were displayed using the EEGLAB toolbox (Delorme and Makeig 2004); see: http://sccn.ucsd.edu/eeglab/). For group-level summary statistics of the individual peak amplitudes we estimated the 95% confidence interval for the difference between straight-ahead and eccentric stimulations using a bootstrap analysis.

*Individual GFP analysis* For all the mean-waves and difference-waves also computed individual global field power (GFP) separately for straight-ahead and eccentric conditions. For all these values, the significance of the difference between the average GFPs for straight-ahead and eccentric stimulations was established by computing its 95% confidence interval (CI) using Bootstrap. Since these data constitute multiple non-independent measures, we applied cluster-based statistics (Pernet et al. 2015). Temporal clustering was formed by contiguous sequences of data points significantly different from zero based on the above-mentioned 95% CI. For each original cluster, we then computed a modified cluster-mass summary statistics based on the sum of the CI lower values. We estimated the null-distribution of the cluster-mass values from 1000 permutations of conditions in the subject GFPs, computation of cluster-masses and storing the maximal cluster-mass values obtained by chance for each permutation. Then we sorted these values and identified the 0.95 quantile. If the cluster-masses of the original clusters were greater than this cluster-based threshold, we qualified the temporal cluster as significant with a *p* value lower than *p* < 0.05.

*Additional EEG analysis using SPM* To complete our analyses and make sure that the effects we found were independent of the chosen methodological approach, we also performed an exploration of our data using the “Statistical Parametric Mapping” software (SPM12; Wellcome Trust Centre for Neuroimaging, University College London, UK; www.fil.ion.uce.ac.uk/spm) implemented in MATLAB (Mathworks Inc., Sherborn, MA). This was done on the pre-processed EEG recordings. We first down-sampled the epochs by 10 for optimizing the analysis rate and truncated the trials between − 100 and + 200 ms around the stimulus onset. For each subject, the data corresponding to our two conditions (straight-ahead or eccentric stimulations) were then converted into 3D images of 32*32*61 voxels where the voltages from all the electrodes at a certain time-point were distributed and interpolated within a 32*32 pixel plane. The third dimension corresponded to the signal time-course during the 300 ms (61 time-points) of the truncated data. To characterize the effects that were common to our two experimental conditions, we performed a second level parametric two-sample T-test, independent samples, equal variance (*p* < 0.05 family-wise error rate, FWER). Significant differences between responses to straight-ahead versus eccentric stimulations were estimated through paired *t* tests (cluster-forming threshold at the significance of *p* < 0.001 uncorrected, while at the cluster level the significance criterion was *p* < 0.05 FWER). To complete the analyses described in the three previous sub-sections, we also characterized our EEG responses on single electrodes and on the cluster of electrodes. The associated results are presented and discussed in the supplementary materials (see Supplementary Fig. 2 and the accompanying text).

*Alpha power spectral analysis* It was previously shown that gaze direction could modulate spontaneous EEG activity in the alpha band (De Toffol et al. 1992). To test whether such effect is related to a straight-ahead preference, we performed a Fourier analysis of our EEG time-courses. This was done for each electrode during the baseline period (i.e. between 600 and 0 ms before stimulus onset) using the absolute single-sided fast Fourier transformation weighted by the sample length. In our study, to avoid contaminations from the preceding responses, we chose a 600 ms time-window for this estimation (it is around a half of the smallest possible interstimulus interval (1000 ms). This time-window still permits to robustly estimate the brain oscillatory activity in the alpha frequency band. We subsequently computed the power spectrum at 10 Hz as a function of gaze direction. To limit the influence of inter-individual variability, the alpha power estimated at each electrode was mean-centered and normalized by the scalp maximum to minimum range. This normalized alpha power is defined as follows:

$$\begin{aligned} &{\text{Alpha}}\_{\text{norm}}\left( i \right) \\&\quad= \left( {{\text{ Alpha }}\left( i \right) - < {\text{Alpha}} > } \right)/\left( {\hbox{max} \left( {\text{Alpha}} \right){-}\hbox{min} \left( {\text{Alpha}} \right)} \right), \end{aligned}$$

where Alpha (*i*) is the alpha power at electrode *i*, <Alpha> is the average alpha power across all electrodes and max (Alpha) and min (Alpha) are, respectively, the maximum and minimum alpha power values across all electrodes. For each subject, these normalized alpha powers were then converted into 32*32 pixel 2D images and analyzed using the SPM approach described in the previous section. This method permits to determine the spatial pattern of significant gaze-driven alterations in alpha power across participants. We subsequently performed a second level parametric one-sample *F* test and a post hoc one sample *T* test in predefined electrodes of interest. Significant effects of gaze fixation were estimated under cluster-forming threshold at the significance of *p* < 0.001 uncorrected, while at the cluster level the significance criterion was *p* < 0.05 FWER. To complete this frequency analysis, we also characterized our EEG responses before stimulus onset in the time domain. The associated results are presented and discussed in the supplementary materials (see Supplementary Fig. 3 and the accompanying text).

## Results

This study aimed at characterizing whether the egocentric position of a visual target affects the event-related potentials (ERPs) recorded in EEG. In particular, we wanted (1) to determine if there is a privileged cortical processing of the straight-ahead direction, as previously reported in publications based on single-cell recordings in macaque (Durand et al. 2010) and neuroimaging data in human (Strappini et al. 2015) and (2) to exploit the high temporal resolution of EEG to characterize the dynamics of the underlying neural mechanisms. Our two experiments were designed to elicit strong visual ERPs with components that corresponded to those that are usually reported in EEG vision studies.

### EEG responses to visual stimulations along the horizontal meridian

**Fig. 2:**
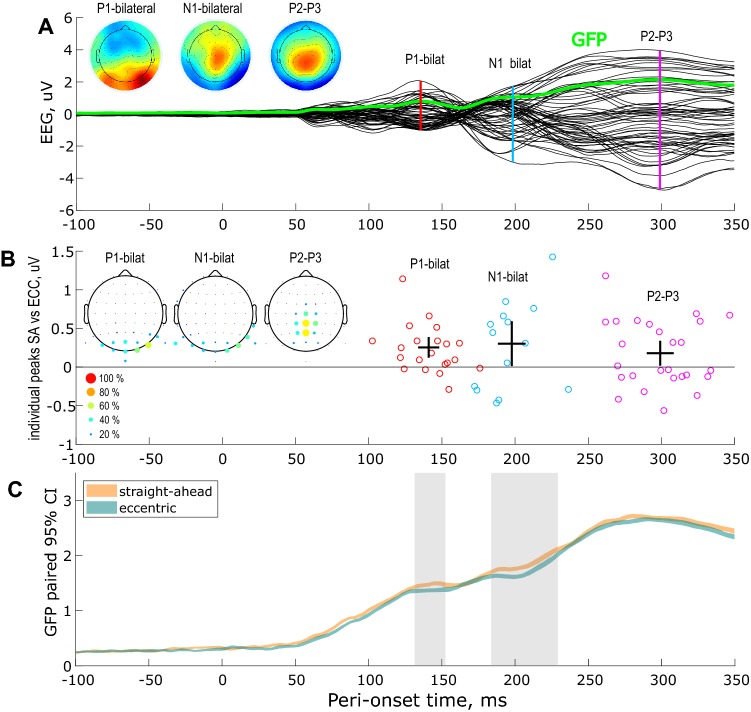
EEG responses to visual stimulations along the horizontal meridian and their facilitation by straight-ahead direction. Mean wave (*n* = 29 subjects). **a** Grand average ERP across subjects, retinal positions and conditions. The corresponding Global Field Power (GFP) is shown in green. The peaks of the three components of interest (bilateral P1 and N1 and central P2–P3) are marked by the vertical color lines. The associated topographic maps are shown on the leftward insets. **b** Individual amplitudes and latencies of the components of interest. The crosses give the 95% confidence interval for both the latencies (horizontal parts) and the amplitudes (vertical parts) of those peaks. The leftward topographies provide the electrode utilization frequency in the estimation of those peaks across subjects, shown as a percentage in color. **c** The average GFPs for straight-ahead (orange) and eccentric stimulations (pale green) was established by computing its 95% confidence interval (CI). Zones of significance for the condition effect are labeled in gray shadow

In the first experiment, we analyzed the ERPs to visual stimuli displayed along the horizontal meridian (either to the left of to the right of ocular fixation) in 29 subjects. Figure 2a shows the grand average (across subjects, across the left and right retinal positions and across the straight-ahead and eccentric conditions) ERP in black. The green time-course corresponds to the associated Global Field Power (GFP). From these time-courses, we can extract three components: (1) the positive, bilateral and occipital P1 that peaks around 140 ms after stimulus onset, (2) the negative, occipital and bilateral N1 that peaks around 198 ms and (3) the positive and central P2-P3 that peaks around 299 ms (Fig. 2b). Those latencies are, respectively, marked by red, blue and magenta vertical lines in the figure. They were used to define time-windows of interest that constrained our research of the components corresponding to straight-ahead and eccentric stimulations at the individual level (see the materials and methods).

The components were extracted when the signal-to-noise ratio (SNR) was sufficient to permit a robust estimation of the peak (see the materials and methods). It was the case in 21 subjects for the bilateral P1 (3.3 μV of average amplitude across these subjects), in 14 subjects for the bilateral N1 (4.7 μV) and in 26 subjects for the central P2–P3 (5.6 μV). For these subjects, the differences in amplitude between straight-ahead and eccentric stimulations are shown in Fig. 2b at the average latencies across these two conditions. Straight-ahead amplitude enhancement of the P1, N1 and P2/3 components were, respectively, equal to 0.2 μV (7% of increase), 0.3 μV (7%) and 0.2 μV (3%).

The electrode utilization frequency in the definition of those peaks across subjects is shown in the leftward topographies (Fig. 2b). The crosses indicate the 95% bootstrap confidence interval for these amplitude difference and average latencies at the population level. We can observe that straight-ahead stimulations led to significantly stronger amplitudes than eccentric stimulations for these three components, as the lower bounds of the associated confidence intervals are greater than 0. To complete our analyses, we also look for differences between the latencies associated with our two conditions. We did not find significant effects in this case as the latencies for straight-ahead and eccentric stimulations were generally in the same range.

As an additional analysis, we compared the GFPs between our two conditions (see Fig. 2c). We found two time-windows during which straight-ahead stimulations led to stronger GFPs than eccentric stimulations. Those time-windows are outlined in pale gray and overlap well with the bilateral P1 and N1 components reported above. This analysis did not permit to define a time-window with significant differences between our two conditions around the peak of the P2–P3 component. This is mostly due to the important variability of this component latency across subject (see Fig. 2b). Overall, our results demonstrate that straight-ahead stimulations lead to stronger ERP amplitudes as early as 140 ms after stimulus onset (i.e. around the bilateral P1 component).

**Fig. 3:**
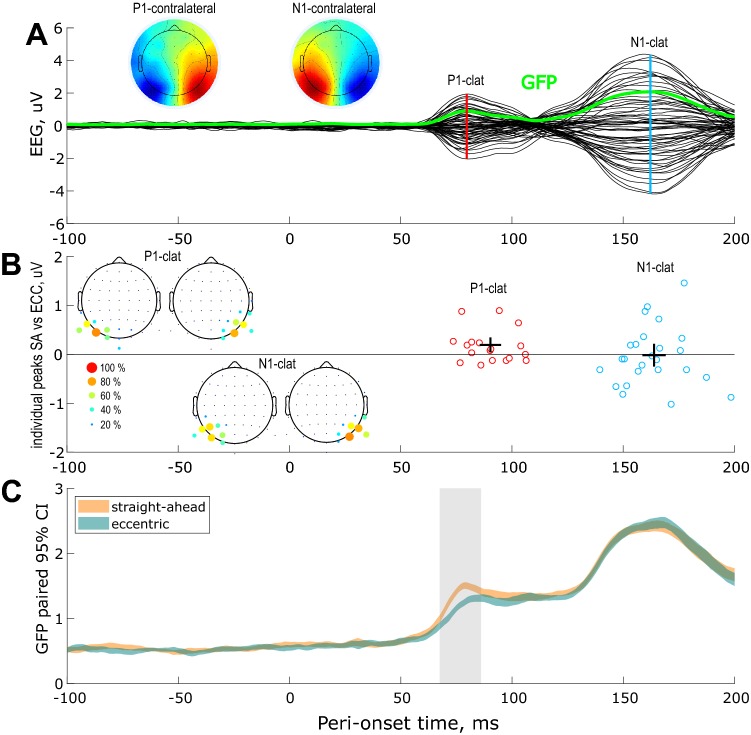
EEG responses to visual stimulations along the horizontal meridian and their facilitation by straight-ahead direction. Difference wave for left vs right visual field (*n* = 29 subjects). **a** Grand average differential ERP across subjects and conditions. The corresponding Global Field Power (GFP) is shown in green. The peaks of the two components of interest (contralateral P1 and N1) are marked by the vertical color lines. The associated topographic maps are shown on the leftward insets. **b** Individual amplitudes and latencies of the components of interest. **c** Straight-ahead effect estimated from the differences of the individual GFPs. Time-windows passing the 95% confidence intervals using bootstrap analysis are outlined in pale gray. See the description in Fig. 2 for more details

To determine whether earlier effects could be detected in our data, we computed the grand average difference-wave ERPs across subjects and conditions corresponding to left vs right retinal stimulations (see the materials and methods). This subtraction permits to discard contributions from overlapping ERPs and thereby to define two additional components that were previously shown to peak around 80 and 160 ms after stimulus onset: the contralateral P1 and N1 (Luck 2012). The grand average differential ERP is shown in Fig. 3a following the same convention as in Fig. 2a.

In our time-courses, the contralateral P1 and N1 peak around 80 and 162 ms, respectively (see the color lines). Based on these latencies, we were able to define individual peaks in 18 subjects for the contralateral P1 and in 25 subjects for the contralateral N1 (see Fig. 3b). Statistical analysis at the individual level showed that straight-ahead stimulations led to stronger contralateral P1. This effect in early contralateral P1 was 0.16 μV on average (6%). This result was confirmed by our GFP analysis (see Fig. 3c) that detected significant difference as early as 68 ms and for a duration of around 18 ms. We did not find significant differences between amplitudes in our two conditions at the level of the contralateral N1 component. Contralateral P1 and N1 latencies for straight-ahead versus eccentric stimulations were not significantly different neither.

### EEG responses to visual stimulations in the quadrants

To investigate whether the straight-ahead direction could affect the earliest measurable EEG responses from primary visual cortex (i.e. from areas V1, V2 and V3), we ran a second experiment in another group of 29 subjects. In this case, stimuli were displayed either in the upper or in the lower visual field (see Fig. 1b and the materials and methods section) so as to elicit strong responses with opposite polarities from neural populations along the lower and upper banks of the calcarine sulcus. The subtraction between EEG responses to these top versus bottom stimulations permits to optimize the extraction of two components, C1 and C2, that peak around 70 and 130 ms after stimulus onset, respectively (Di Russo et al. 2003; Miller et al. 2015).

**Fig. 4:**
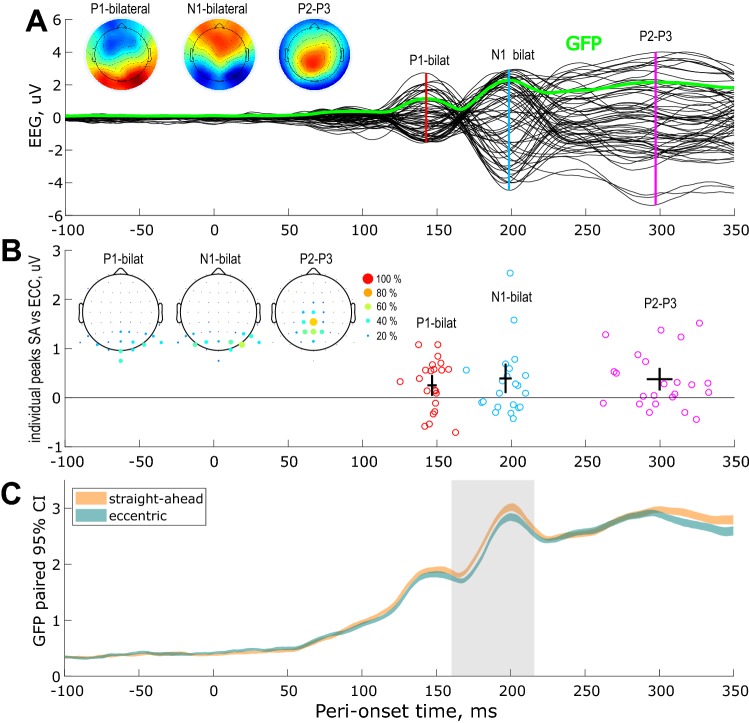
EEG responses to visual stimulations in the quadrants and their facilitation by straight-ahead direction. Mean wave (*n* = 29 subjects). **a** Grand average ERP across subjects, retinal positions and conditions. The corresponding global field power (GFP) is shown in green. The peaks of the three components of interest (bilateral P1 and N1 and central P2-P3) are marked by the vertical color lines. The associated topographic maps are shown on the leftward insets. **b** Individual amplitudes and latencies of the components of interest. **c** Straight-ahead effect estimated from the differences of the individual GFPs. Time-windows passing the 95% confidence intervals using bootstrap analysis are outlined in pale gray. See the description in Fig. 2 for more details

Before the difference-wave analysis on these specific components, we controlled as a proof of robustness that the effects observed in our first experiment were also detectable in this second dataset. We, therefore, computed the mean-wave grand average (by pooling across subjects and experimental conditions) ERP and reproduced the analyses described above. The results of this analysis are shown in Fig. 4. At the individual level, the bilateral P1 and N1 components were measurable in 22 subjects. The central P2-P3 was obtained in 23 subjects. As in the first experiment, the amplitudes of these peaks were significantly stronger for straight-ahead stimulations (see the 95% confidence interval for the difference between conditions in Fig. 4b). Straight-ahead stimulations, respectively, enhanced the amplitudes of the P1, N1 and P2/3 by 0.3 μV (7% of increase), 0.3 μV (5%) and 0.3 μV (3%).

The GFP analysis confirmed these results for the bilateral N1 (see Fig. 4c). GFP for straight-ahead responses were also stronger around the peak of the bilateral P1 but the difference with GFP for peripheral stimulations did not reach significance. This absence of significance might be explained by the greater heterogeneity in stimulus positions for this second experiment. In general, latencies and amplitudes of all detected components parallel those of the first experiment.

**Fig. 5:**
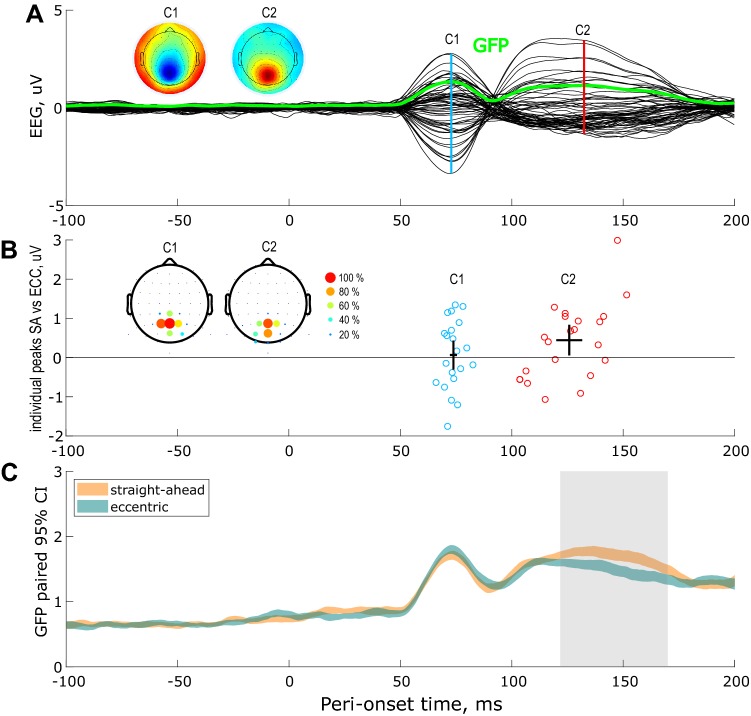
EEG responses to visual stimulations along the horizontal meridian and their facilitation by straight-ahead direction. Difference wave for upper vs lower visual field (*n* = 29 subjects). **a** Grand average differential ERP across subjects and conditions. The corresponding global field power (GFP) is shown in green. The peaks of the two components of interest (C1 and C2) are marked by the vertical color lines. The associated topographic maps are shown on the leftward insets. **b** Individual amplitudes and latencies of the components of interest. **c** Straight-ahead effect estimated from the differences of the individual GFPs. Time-windows passing the 95% confidence intervals using bootstrap analysis are outlined in pale gray. See the description in Fig. 2 for more details

Then we computed across subjects and conditions the grand average difference-wave ERPs for upper vs lower visual field stimulations. Upper grand average ERP was the mean of upper-left and upper-right retinal stimulations while lower grand average ERP was the mean of lower-left and lower-right retinal stimulations (see the materials and methods). This method permits to assess the C1 and C2 components (Di Russo et al. 2003, 2012; Miller et al. 2015). The grand average differential ERP is shown in Fig. 5a following the same convention as in the previous figures.

In these time-courses, the C1 and C2 components peaked at 73 and 130 ms after stimulus onset and had occipital topographies, in agreement with previous reports (Di Russo et al. 2012). At the individual level, the C1 component was measurable in 21 subjects and the C2 in 22 subjects (see the materials and methods). We did not find any significant difference between straight-ahead and eccentric stimuli at the level of the C1 component. However, our bootstrap analysis revealed that the C2 amplitude was significantly stronger for straight-ahead than for eccentric stimulations (see the corresponding confidence interval in Fig. 5b). Straight-ahead stimuli produced an increase in the individual amplitudes of the C2 equal to 0.41 μV (10%).

GFP analyses confirmed these results by showing that the earliest difference between our two conditions emerged around 130 ms after stimulus onset, i.e. around the peak of the C2 component (see Fig. 5c). As in all previous analyses, we did not detect any significant differences between the peak latencies in our two conditions. In this second experiment, the earliest measurable EEG responses were, therefore, unaffected by the spatial position of the stimulus. The first observable straight-ahead effects were observed for the bilateral P1 component around 150 ms after stimulus onset.

**Fig. 6:**
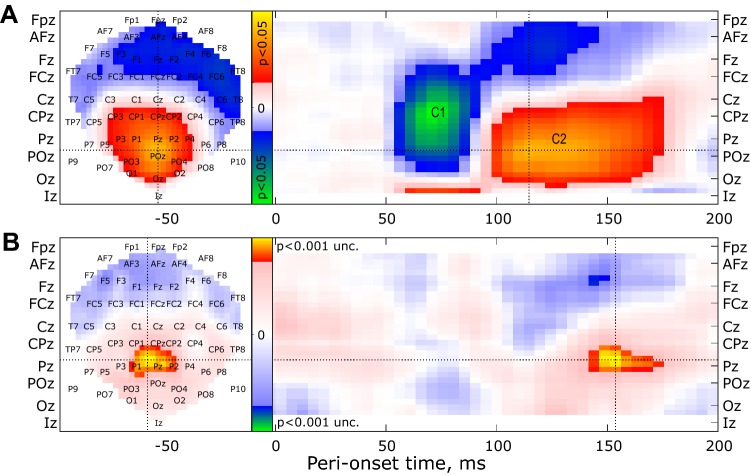
Spatio-temporal analysis of the significant differences between upper versus lower (**a**, top row) and between straight-ahead versus eccentric (**b**, lower row) stimulations. The left and right panels, respectively, provide the spatial and temporal distribution of the *T*-scores. Data were thresholded to highlight significant clusters (*p* < 0.05, FWER in the upper panel and *p* < 0.001, uncorrected in the lower panel)

Spatio-temporal consistency of C1/C2 components across subjects allowed us to perform an additional analysis in SPM (see the ‘Materials and methods’ section). In Fig. 6, the first row shows spatio-temporal distribution of the *T*-scores for the differences between stimulations in the upper versus lower visual field (thresholded at *p* < 0.05, FWER). Significant differences are observed in parieto-occipital electrodes with polarities and latencies that correspond to the C1 and C2 components, in agreement with the results shown in Fig. 5. The second row shows the spatio-temporal distribution of the *T*-scores for the differences between straight-ahead versus eccentric stimulations (thresholded at *p* < 0.001, uncorrected). The earliest significant effects are observed around 150 ms after stimulus onset and are generated in centro-parietal electrodes. It matches well with the enhanced responses to straight-ahead stimulation shown in Fig. 5. Here as well, we did not find significant straight-ahead effects at earlier latencies. This result, obtained through different approaches, confirms that in our experience, straight-ahead stimulations did not lead to measurable modifications of the C1 component.

### Alpha power spectral analysis

**Fig. 7:**
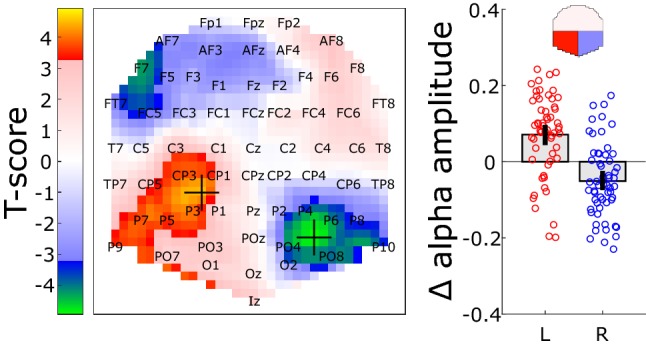
Effects of gaze direction (rightward versus leftward gaze) on the alpha power during the 600 ms pre-stimulus baseline (experiments 1 and 2). The statistical parametric map (leftward panel) provides the spatial distribution of the *T*-scores. Data were thresholded to highlight significant clusters (*p* < 0.05, FWER). The rightward panel shows the effect across all the left (L) and right (R) occipito-parietal electrodes (see the red and blue regions on the upper topographic map). Bars and whiskers provide the means and associated 95% confidence intervals. Each dot corresponds to an individual subject

A previous study showed that gaze direction could modulate spontaneous EEG activity in the alpha band (De Toffol et al. 1992). To test whether the same effects are observable in our measurements and also whether they can be related to the straight-ahead direction, we performed a Fourier analysis of the 600 ms pre-stimulus baseline (see the ‘Materials and methods’ section). Because this analysis is based on single-trials and, therefore, more susceptible to noise, it was done on all the data from experiments 1 and 2 to increase the statistical power. Figure 7 shows the difference in pre-stimulus alpha power for a rightward versus a leftward gaze.

Significant differences are observed in the left and right occipito-parietal cortex (see the leftward panel). The rightward panel provides the average values and associated 95% intervals for all the left (L) and right (R) occipito-parietal electrodes (see the red and blue boxes in the leftward panel). Each dot corresponds to one individual subject. Note that here, we preferred to group electrodes together rather than to explore the effects at the maxima of the statistical analysis to avoid the ‘double dipping’ that arises when the same data are used both for identifying sites of interest and for characterizing their activity. The difference between effects in left versus right occipito-parietal electrodes is highly significant (*p* < 0.001, permutation tests) and reflects a reduction in alpha power in the hemisphere ipsilateral to gaze direction (and thus contralateral to the straight-ahead direction). We also analyzed the data from experiments 1 and 2 separately and found significant effects for experiment 1 (*p* < 0.001) as well but only a trend for experiment 2 (*p* = 0.075). This difference might reflect an additional effect of stimulus position, e.g. via expectation mechanisms. Altogether, this analysis confirms that gaze direction affects pre-stimulus preparatory activity and suggests that this modification could be directly related to the straight-ahead direction, as consistent alpha power reductions are found in the hemisphere contralateral to this spatial position.

### Behavioral responses

The average RT did not differed between the two experiments. The group medians and 95% confidence intervals were, respectively 344 [330:356] and 345 [331:355] ms for the first and second experiment. The RT difference between the straight-ahead and eccentric condition was not significant in both experiments: +1 [− 1:2] ms and 0 [− 1:2] ms.

## Discussion

The aim of this study was to characterize the dynamics of the privileged processing of the straight-ahead direction in human visual cortex. We recorded the EEG responses to peripheral stimuli that were visually identical but that were presented either straight-ahead or at eccentric positions (Fig. 1). Our experiments both demonstrated that straight-ahead stimulations lead to stronger P1, N1 and P2–P3 components (Figs. 2, 4). We did not observe any significant modifications in the latencies of these components. It suggests that the straight-ahead preference is characterized by a gain augmentation of the responses, in agreement with fMRI recordings in human (Strappini et al. 2015), single-cell recordings in macaque V1 (Durand et al. 2010) and more generally with several studies on gaze position effects on neural activations in primate, see, e.g. (Trotter and Celebrini 1999; Merriam et al. 2013). In addition, our frequency analysis of pre-stimulus brain activity suggests that the straight-ahead direction is associated with reduced alpha power in the contralateral hemisphere before stimulus onset (see Fig. 7). Previous EEG studies showed that pre-stimulus alpha power suppression is associated with enhanced evoked activity (Bacigalupo and Luck 2019; van Dijk et al. 2008) and modifications of the P1 amplitude (Fellinger et al. 2011). Our observations are also in line with the pre-stimulus preparatory activity found in the occipital areas for simple reaction tasks by Di Russo et al. (2019). The modulation of the evoked response that we observed for straight-ahead stimuli could, therefore, be linked to this pre-stimulus and gaze related alpha suppression.

In our study, the latencies of the P1 and N1 components are in very good agreement with those measured with peripheral stimulation (Novitskiy et al. 2011; Di Russo et al. 2002). These latencies are slightly longer than those triggered by foveal stimulation, e.g. (Di Russo et al. 2019). Peripheral stimuli are indeed known to evoke slower EEG responses (Hansen et al. 2016). Because the mean wave might reflect a mixture between the C1 (peak latency of 70 ms) the contralateral P1 (peak latency of 80 ms) and the bilateral P1 (peak latency of 140 ms), it could have introduced some variability in the peak latencies we observed (see Fig. 2). To cancel out this variability, we performed additional analyses based on differences between waves. The contralateral P1 was estimated from the difference between responses to left versus right stimulations and the C1 and C2 components were estimated from the difference between upper versus lower stimulations. On the obtained difference waves, individual latencies and topographies were more consistent, which permitted a more robust characterization of the ERP components at the individual level.

Our analysis did not allow us to well discriminate between the P2 and P3 components from the mean wave, probably because they have similar topography and very close latencies in simple reaction time tasks (Di Russo et al. 2019). We, therefore, cannot be sure whether the strongest EEG responses that we obtained for these components during straight-ahead stimulation correspond to the P2 and/or the P3.

Overall, the earliest effects that we measured arose around 70 ms after stimulus onset and correspond to the contralateral occipital P1 (Fig. 3). Previous studies based on EEG source localization approaches combined with neuroimaging data proposed that this component is generated in extra-striate visual cortex, first of all in dorso-occipital areas (V3, V3A) and then in ventro-occipital regions (V4) (Di Russo et al. 2001, 2003; Martinez et al. 1999).

Our second experiment was specifically designed to determine whether this privileged processing of the straight-ahead direction can be detected in the first measurable EEG responses from early visual cortex (i.e. from areas V1, V2 and V3). We confirmed here the results of the first experiment, and, in addition, after the subtraction between ERPs to upper versus lower visual hemifield stimulations, we obtained strong C1 and C2 components (Fig. 5) whose amplitudes and latencies are very consistent with previous studies (Di Russo et al. 2003; Miller et al. 2015). With this approach, gain modulations were first observed around 130 ms after stimulus onset, which corresponds to the C2 component. We confirmed that this result was not caused by our methodological approach with an additional analysis that led to the same conclusion (Fig. 6). Because of its latency and its polarity reversal for top versus bottom stimulation, this component is believed to reflect feedbacks from higher-level visual areas to the early visual cortex (Miller et al. 2015). It was previously suggested that these feedbacks might provide information about low and high-level scene features, such as the category or the depth of the stimuli (Morgan et al. 2016; Revina et al. 2018). That top–bottom feedback signals are thought to modulate sensory input, providing information about the global scene structure. Recent models of dynamic predictive updating also proposed that these top-down signals could inform about the global structure of the visual scene (Edwards et al. 2017). Our results are in line with these studies and demonstrate that neural processing in human primary visual cortex is not purely retinotopic and that it integrates egocentric spatial properties.

In our data, the first detectable modulation of the evoked responses for straight-ahead stimuli occurred for a component whose possible cortical generators are extra-striate areas. One previous paper characterized the influence of eye position on EEG recordings using two electrodes (Andersson et al. 2004). In this study, gaze direction modulated the amplitude of the C1 component but at relatively late latencies that ranged between 94.8 and 98 ms. However, these authors only measured the responses to stimulations in the lower visual field and were, therefore, not able to compute the difference between ERPs in response to upper versus lower stimulation as in the present study. With their approach, the C1 and P1 components overlap and become difficult to disentangle (Luck 2005). Given the latency that they reported, the gaze effect in their study was most likely driven by the P1 component and is, therefore, in agreement with our results.

Our data demonstrate that straight-ahead effects become significant in early visual cortex around 130–160 ms after stimulus onset. This is compatible with previous single-cell recordings performed by our group which showed that the spike rate of V1 neurons is further increased for straight-ahead stimulations 100–150 ms after stimulus onset (Durand et al. 2010). If small incongruences may exist between the two studies (e.g. the first straight-ahead effects are measured earlier in macaque), it remains difficult to determine whether they arise from methodological and/or specie differences. Additional experiments are required to clarify this point, for example using intracranial recordings in implanted epileptic patients, see (de Jong et al. 2016).

Can the straight-ahead preference be related to attention mechanisms? In our previous behavioral study (Durand et al. 2012), a dual task at fixation did not alter the straight-ahead effects. Gain modulation for straight-ahead stimulations was also observed in an fMRI study performed under passive viewing conditions (Strappini et al. 2015). However, these manipulations did not require full attentional resources and, therefore, do not rule out an attentional explanation (see the discussion in Strappini et al. (2015). Indeed, several ERP experiments showed that the P1, N1 and P2 components are affected by spatial attention (Hillyard and Anllo-Vento 1998). Van Voorhis and Hillyard notably suggested that these attention effects could arise in the early phase of the P1 at a latency (65 ms after stimulus onset) that is consistent with the timing of our earliest straight-ahead effect on the evoked responses (at 68 ms) (Van Voorhis and Hillyard 1977). Moreover, a growing number of studies proposed that spatial attention leads to alpha desynchronization (i.e. to a decrease in alpha power) in the ‘attended’ hemisphere (see e.g. Kelly et al. (2006), as the straight-ahead direction in our study. Finally, before stimulus onset, we also observed consistent gaze-dependent drifts in frontal cortex that might correspond to pro-active attention mechanisms (see Supplementary Fig. 3 and the accompanying text). Altogether, these findings suggest that the privileged processing of the straight-ahead direction might be driven by neural mechanisms close to those involved in spatial attention and possibly triggered by proprioceptive signals related to the position of the eyes in their orbit.

In a recent study, we showed that straight-ahead stimuli triggered faster saccades than stimuli in eccentric space (Camors et al. 2016). The straight-ahead amplification observed for the P2 component in the present study is in line with a cortical mechanism that would facilitate action preparation. Indeed, besides other functions, an important process that affects the P2 amplitude is cross modal visuo-tactile integration. Vision of the hand enhances the tactile P2 (Torta et al. 2015) and the vertex P2 potential is also stronger for congruent visuo-tactile events (Longo et al. 2012). Straight-ahead effects on the P2 could, therefore, in addition to attention mechanisms, could reflect visuo-motor integration that facilitates interaction with objects located in front of the body.

By contrast with our previous report (Durand et al. 2012), we did not find significantly shorter reaction times for straight-ahead stimuli in the present study. Overall, our reaction times were significantly longer (350 ms against 300 ms), despite the fact that we used larger (~ 6°*10° against 2° in diameter) and more contrasted (100% against 30%) stimuli to evoke strong ERP components. Both the extension of the reaction times and the absence of a straight-ahead effect might have their root in the fact that the stimuli were located within the peri-personal space in our previous study (40 cm), while they were farther away (150 cm) in the present experiments. Since peri-personal space is known to be associated with behavioral facilitation (Graziano and Cooke 2006) and to trigger shorter reaction times (Li et al. 2011), we hypothesize that the behavioral straight-ahead facilitation measured by simple reaction time might occur only within the peri-personal space.

When presented in eccentric position, stimuli had small vertical disparity because they were closer to one eye than to the other. In our previous studies (Durand et al. 2010, 2012), we showed that under monocular viewing, straight-ahead effects remained unchanged. Moreover, vertical disparities become negligible at long viewing distances such as the one used in this study. It is, therefore, unlikely that vertical disparity had an impact on our results. Poor gaze fixation with a drift towards the center could be another confound, but we controlled gaze fixation with an eye-tracker in a subset of subjects and did not find any significant differences between fixation during our straight-ahead and eccentric conditions (see supplementary materials file). Such a centripetal bias would also modify the retinotopic positions of our stimuli and alter the C1 but we did not measure such a change. Finally, we used a curved screen so that straight-ahead and eccentric stimuli were localized at the same distance from the subjects. It excludes distance as the cause of our results.

To conclude, our EEG data confirmed previous reports showing that the straight-ahead direction is preferentially processed in the humans (Strappini et al. 2015) as it is the case in non-human primates (Durand et al. 2010). The earliest measurable effects on the evoked responses appeared around 70 ms after stimulus onset. Alpha power suppression in the hemisphere contralateral to the straight-ahead direction was also observed before stimulus onset. Altogether, the neural mechanisms described here demonstrate that visual processing, even in its early phases, is not uniquely retinotopic and also reflects egocentric properties. They, therefore, impose important modifications on current models of vision and spatial perception.

## Electronic supplementary material

Below is the link to the electronic supplementary material.

Supplementary material 1 (DOCX 839 kb)
