# Supplementary material for: Dynamics of the straight-ahead preference in human visual cortex
Source: Brain Struct Funct. 2019 Dec 2;225(1):173–86. doi: 10.1007/s00429-019-01988-5 (PMC6957552; doi:10.1007/s00429-019-01988-5)
Supplement: Supplementary file 1 — Supplementary material 1 (DOCX 839 kb) [file 429_2019_1988_MOESM1_ESM.docx]

**Supplementary materials**

For the paper ‘Dynamics of the straight-ahead preference in human visual cortex’

*Olena V. BOGDANOVA, Volodymyr B. BOGDANOV, Jean-Baptiste DURAND, Yves TROTTER and Benoit R. COTTEREAU*

1. **Analysis of the participants’ eye movements**

We recorded eye movements in 10 subjects from experiment 2 with an eye tracker (Eye Link 1000 desktop) placed 35 cm in front of the participants. The sampling rate was 500 Hz. We first estimated the distribution of gaze fixations for each session of each subject. We then convolved the two-dimensional matrix of cumulative fixation scores with a Gaussian kernel of 1° (0.25° sigma). We defined the local maxima for the gaze left and right conditions (see the leftward panel on **Supplementary figure 1**). The distribution around these peaks was estimated for circular regions of interest with a radius ranging from 0.1° to 10° by step of 0.1° in order to find the critical radius that included 95% of gaze samples. The median value of this critical radius was then computed for each subject across sessions. The average of these median values across subjects (and the associated 95% confidence interval) is shown on the rightward panel of **Supplementary figure 1**. On average, 95% of fixations were located within a 1.4° radius zone around the 2 fixation points. In order to determine whether subjects slowly drifted their gaze toward the straight-ahead direction, we compared gaze position between the first and last quarters of each 30 s block of leftward and rightward fixations. The corresponding 95% confidence intervals were [-0.08 : 0.09 degrees] and [-0.08 : 0.07 degrees] where positive values correspond to a rightward drift. They show that gaze fixation remained very stable during our recordings and therefore that the differences we obtained between the EEG responses to straight-ahead versus peripheral stimulations are not likely to be caused by differences in retinal eccentricity.

| 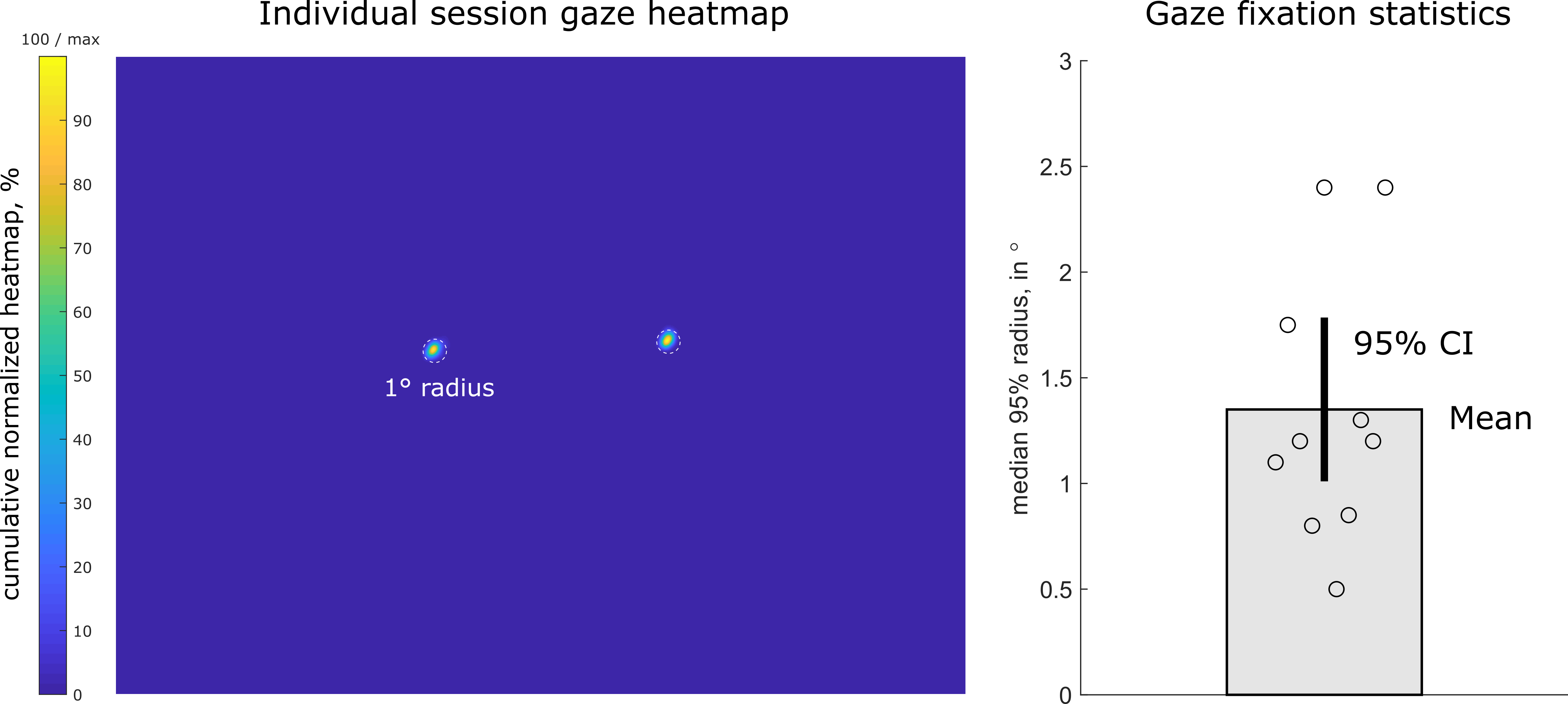 |
| --- |
| **Supplementary Figure 1.** *Gaze fixation data for 10 subjects during the second experiment. The leftward panel shows the cumulative gaze fixation heatmap for one exemplar subject during one session of the second experiment. The rightward panel gives the average median value (n = 10) of 95% cumulative gaze fixation and the associated 95% confidence interval. Each dot corresponds to an individual subject.* |

| **A** | **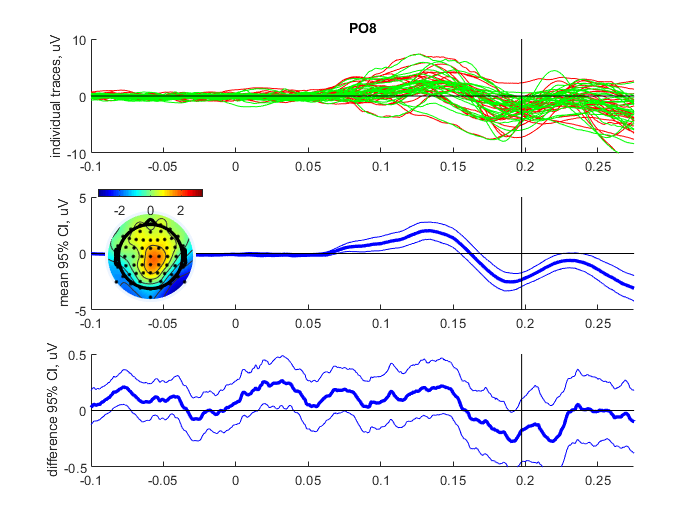** | **B** | **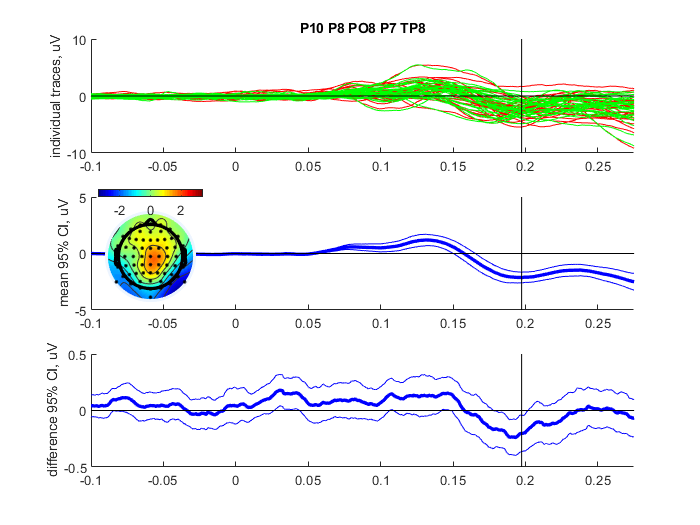** |
| --- | --- | --- | --- |
| **C** | 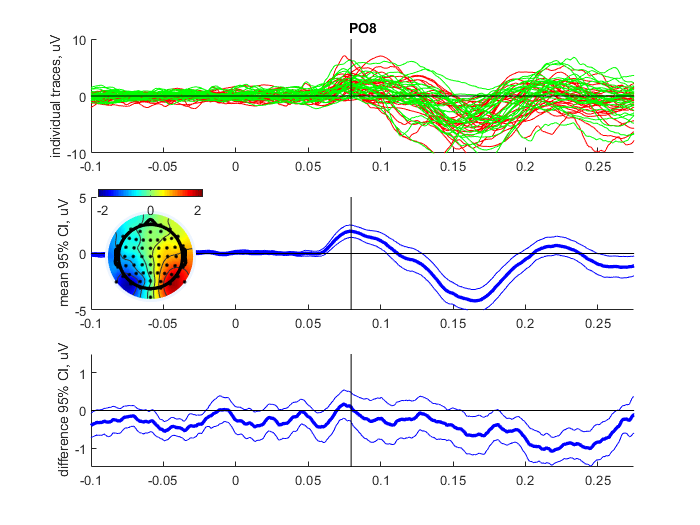 | **D** | **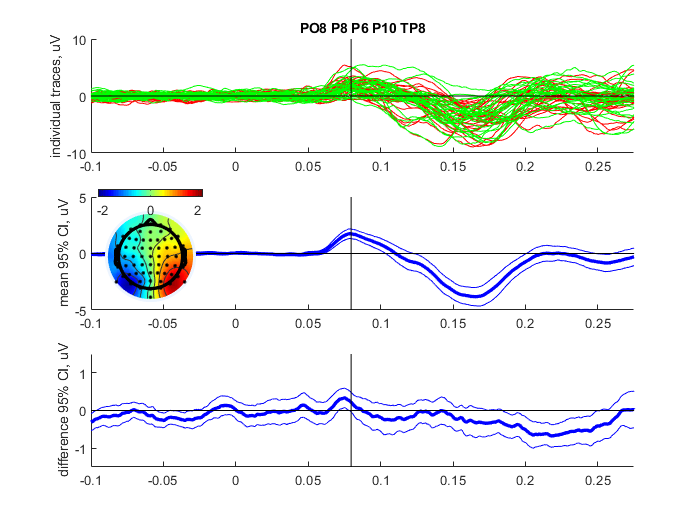** |
| **E** | **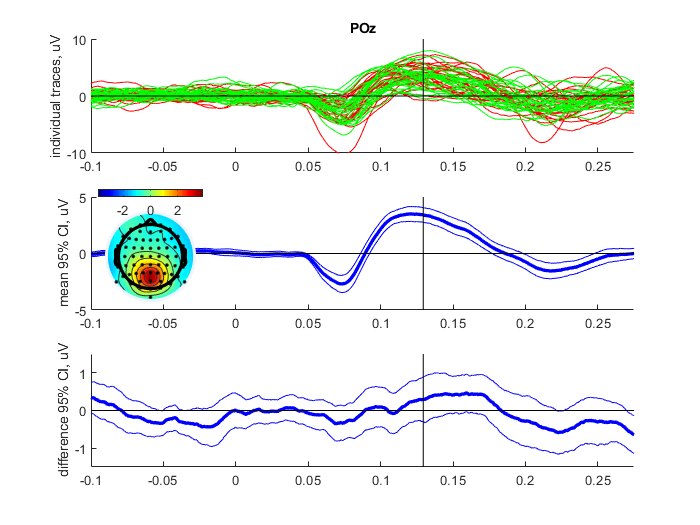** | **F** | **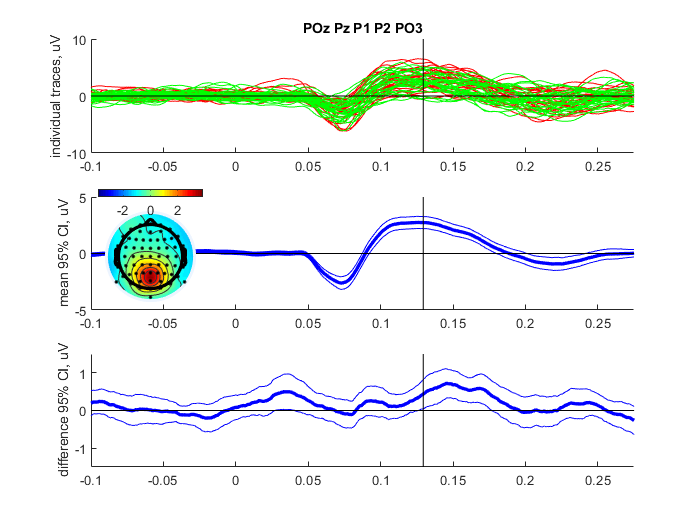** |
| **Supplementary Figure 2.**  ERP *analyses for single representative electrodes (A, B, C) and clusters of electrodes (D, E, F) selected from their maximum amplitude during component-specific time-windows (****vertical black lines*** *provide the peaks of their associated component):*  ***A:*** *Mean wave: peak for the N1 bilateral component (PO8);*  ***B:*** *Mean wave: N1 bilateral significant effect (occipito-bilateral cluster of electrodes);*  ***C:*** *Left-right difference wave: peak for the P1 contralateral component (PO8);*  ***D:*** *Left-right difference wave: P1 contralateral significant effect (occipito-bilateral cluster of electrodes);*  ***E:*** *Top-bottom difference wave: peak for the C2 component (POz).*  ***F:*** *Top-bottom difference wave: C2 significant SA effect (occipito-central cluster of electrodes).*  ***Top:*** *grand average for each subject (n=29). Red and green time-courses respectively provide ERPs in response to straight-ahead and eccentric visual stimulations.* ***Middle:*** *95% bootstrap confidence intervals corresponding to the average time-courses across conditions.* ***Bottom:*** *Confidence intervals for the difference between straight-ahead versus eccentric ERPs.* | | | |

1. **Analysis of evoked potentials on single electrodes and electrodes’ clusters**

To complete the analyses described in the main manuscript, we also computed the event related potentials (ERPs) on single electrodes (**Supplementary Figure 2, A, C, E**) and electrode clusters (**Supplementary Figure 2, B, D, F**). This approach globally confirms the results described in the main manuscript, as difference in amplitudes can be observed between the EEG responses to straight-ahead versus eccentric stimulations. These differences are nonetheless not as robust (see the confidence intervals on panels C) as those obtained with the GFP and/or peak analyses for which effects were very significant across subjects.

1. **Analysis of pre-stimulus preparatory activity**

In order to characterize gaze-dependent pre-stimulus processing in the time-domain, we followed the procedure described in (Di Russo et al., 2019). We performed an analysis of the EEG recordings during the 600 ms directly preceding stimulus onset. This analysis only included trials after an inter-stimulus interval (ISI) comprised between 1600 and 2200 ms. This time window was selected to avoid a possible impact of motor responses from the previous trials in the case of the shortest ISIs (1000 ms). To improve the quality of the analyzed signal, we also filtered out the 40% noisiest trials determined from the highest peak-to-peak amplitudes across electrodes. Following this, we analyzed a gaze-dependent difference in voltage drift between the first and the last 100 ms of the 600 ms pre-stimulus period. This was done separately for leftward and rightward fixations. We then computed the difference between the drifts corresponding to rightward versus leftward gaze fixation. This contrast provides the difference between the preparatory activations corresponding to straight-ahead versus eccentric visual stimulations. Across our two experiments, we found significant and consistent effects in frontal electrodes. During rightward gaze, drifts were relatively more negative in leftward frontal electrodes (see **Supplementary figure 2**) which correspond to the hemisphere contralateral to eccentric stimulations. It also corresponds to the hemisphere where under similar conditions, a significant increase of alpha activity is observed during the 600ms before stimulus onset (**Figure 7 in the paper**). The opposite effect was found for the leftward gaze.


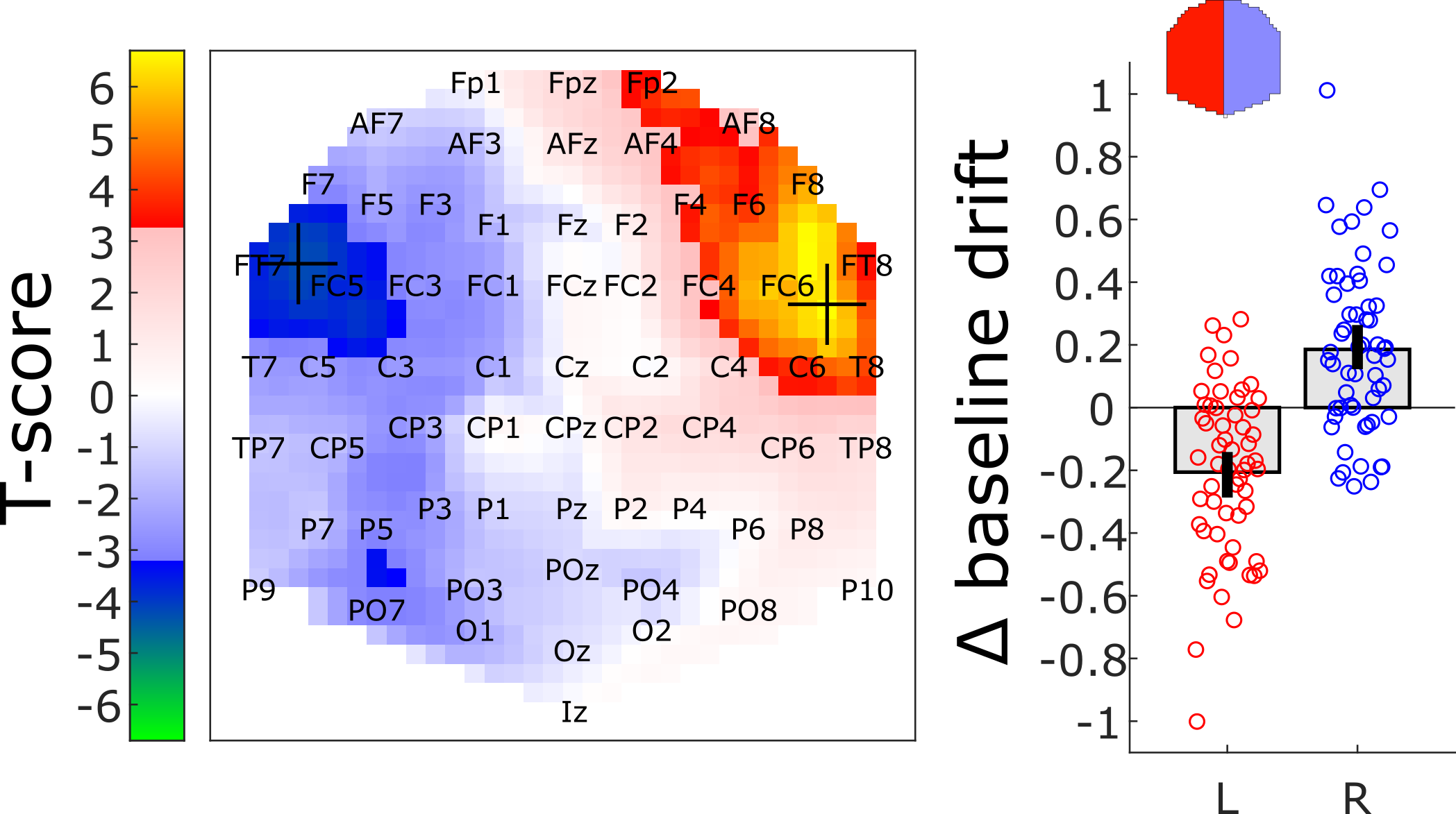
**Supplementary Figure 3.** *Effects of gaze direction (rightward versus leftward gaze) on the voltage drift during the 600ms pre-stimulus baseline for both experiments 1 and 2. The statistical parametric map (leftward panel) provides the spatial distribution of the T-scores. Data were thresholded to highlight significant clusters (p < 0.05, FWER). The rightward panel shows the effect across the left (L) and right (R) hemispheres (see the red and blue regions on the upper topographic map). Bars and whiskers provide the means and associated 95% confidence intervals. Each dot corresponds to an individual subject.*

With this analysis, we did not find significant changes in occipital regions that were consistent across the two experiments, like the vN component described in (Di Russo et al., 2019). It is possible that in our case, time-domain pre-stimulus activity in occipital areas was more affected by the difference in task demands between the two experiments than by gaze-direction. Indeed, stimuli were spatially more scattered and thereby less predictable in our second experiment. The consistent and significant gaze-dependent pre-stimulus drift that we observed in our two groups of subjects in anterior sites can potentially correspond to the pN component (Ragazzoni et al., 2019). This component is associated with cognitive preparation intended as proactive attention in prefrontal cortex (Ragazzoni et al., 2019) which can cause local increase in alpha power on ipsilateral recording sites via descending inhibition. This interpretation is consistent with the strong gaze-dependent alpha power modulation that we observed before stimulus onset (figure 7).

**REFERENCES**

Di Russo, F., Berchicci, M., Bianco, V., Perri, R.L., Pitzalis, S., Quinzi, F., Spinelli, D., 2019. Normative event-related potentials from sensory and cognitive tasks reveal occipital and frontal activities prior and following visual events. Neuroimage 196, 173-187.

Ragazzoni, A., Di Russo, F., Fabbri, S., Pesaresi, I., Di Rollo, A., Perri, R.L., Barloscio, D., Bocci, T., Cosottini, M., Sartucci, F., 2019. "Hit the missing stimulus". A simultaneous EEG-fMRI study to localize the generators of endogenous ERPs in an omitted target paradigm. Sci Rep 9, 3684.
